# Dilemmas of medical overuse in general practice – A focus group study

**DOI:** 10.1080/02813432.2019.1569370

**Published:** 2019-01-31

**Authors:** Per Øystein Opdal, Eivind Meland, Stefan Hjörleifsson

**Affiliations:** a Department of Global Health and Primary Care, University of Bergen, Bergen, Norway;; b Research Unit for General Practice, NORCE Norwegian Research Center, Norway

**Keywords:** Medical overuse, family practice, qualitative research, diagnostic methods, ethics

## Abstract

**Objective:** To obtain first-hand in-depth accounts of overtesting amongst GPs in Norway, as well as the GPs’ perspectives on drivers of overtesting and strategies that can prevent overtesting.

**Design and setting:** Four focus groups with GPs were conducted. All participants were asked to share examples of unnecessary testing from their everyday general practice, to identify the driving forces involved in these examples and discuss any measures that might prevent excessive testing. All authors collaborated on the analysis, conducted as systematic text condensation, using critical incident technique.

**Results:** This study reveals two main positions regarding overtesting in general practice. In the categorical position there is no such thing as overtesting and GPs are obliged to perform extensive investigations on the suspicion that any person can carry a fatal disease, no matter how minor or absent their symptoms are. In contrast, in the dilemmatic position, the GPs acknowledge that investigations can cause significant harm, but still feel pressured to discover disease at the earliest opportunity and to meet patients’ demands. The GPs’ strategies for resolving this dilemma are often demanding and not always successful, but sharing uncertainty and fallibility with patients and colleagues appears to be the most promising strategy.

**Conclusions:** Our study indicates that GPs in Norway experience a strong pressure to discover any instance of disease and to meet patients’ demands for investigations. One way of preventing the harm that accrues from overtesting is openly sharing uncertainty and fallibility with patients and colleagues.

## Introduction

There is a growing awareness of medical overuse amongst governments, service providers, research centres and clinical experts throughout the world. For the purpose of this paper, medical overuse is broadly defined as ‘the provision of medical services for which the potential for harm exceeds the potential for benefit’ [1], and occurs in the form of overtesting and overtreatment [2]. Medical overuse is known to occur in all parts of the world and in all domains of medicine, often resulting in waste and harm, and is correlated with underuse, i.e. people who need medical care not receiving adequate help [3].

In general practice, guidelines and principles that derive from the specialist paradigm are among the drivers of medical overuse, and some authors have called for a bolstering of the generalist expertise of general practitioners (GPs) to curb overuse [4]. In Norway, one of the expressed aims of primary care is for GPs to ensure the prudent allocation of healthcare resources, in line with the WHO vision for primary care [5]. Medical overuse has in recent years been high on the agenda of The Norwegian College of General Practice and in 2016 the college published a position paper on medical overuse [6].

On the other hand, most clinical guidelines in Norway do not address medical overuse, and concerns have been raised that GPs are not performing their intended role as gatekeepers [7]. A Commonwealth Fund survey in 2015 found that only one of three GPs in Norway believed that their patients received too much medical care [8]. However, in 2017, a survey by the Norwegian College of General Practice found that three out of four GPs believe that medical overuse occurs in their own practice (email from Sigrid R⊘d special advisor at the Norwegian Medical Association August 31 2018). Beyond this, Norwegian GPs’ understanding of the role they themselves play with regard to medical overuse has not been explored.

In this study we have looked at the aspect of medical overuse that involves overtesting among Norwegian GPs. The objective was to obtain (1) first-hand in-depth accounts from Norwegian GPs of overtesting in general practice, but also the GPs’ perspectives on (2) drivers of overtesting in their individual domains of health care, and (3) strategies that can help prevent such overtesting. Our personal point of view that motivated us to conduct this study is that overtesting is likely to be a considerable problem in general practice.

## Material and methods

We conducted four focus groups with GPs working in the cities of Bergen and Oslo, and in rural districts close to those cities. The interviews were conducted during the years 2014/2015. Groups one and two (*n* = 9 and *n* = 8) were conducted with trainees who were meeting for a regular session with their trainer as a part of their postgraduate training. Groups three and four (*n* = 5 and *n* = 6) were conducted with experienced and accredited GPs convening for a regular session mandatory for recertification. We deemed four groups to provide sufficient information power for an exploratory analysis, where the goal was to discern relevant patterns in keeping with the study´s aim [9].

Recruitment was by email, which was sent to groups identified by convenience through our own extended professional networks, asking them to contribute to research on overtesting in general practice. All four groups that were contacted by email agreed to participate. Participants also received an additional email, asking them to (1) share examples of unnecessary testing from everyday general practice, (2) identify the driving forces involved in these examples and (3) discuss any measures that could have been helpful in preventing the excessive testing related to their examples. The interview guide consisted of the above three questions and additional follow-up questions to facilitate the sharing of stories and perspectives on the same topics.

Each focus group convened for a single session lasting 1½ hours. The interviews were audio recorded and transcribed verbatim. All authors collaborated on the analysis, conducted as systematic text condensation [10], using critical incident technique [11] to learn from the stories that were shared during the focus groups. The four steps of text condensation comprised of (i) reading all the material to obtain an overall impression, (ii) identifying units of meaning, representing different aspects of the participants' experiences and coding for these, (iii) condensing the contents of each of the coded groups, and (iv) summarizing the contents of each code group to generalize descriptions and concepts.

## Results

This study revealed two main findings regarding overtesting in general practice. The *categorical position*, expressed preferably by trainee GPs, is that there is no such thing as overtesting. Therefore, they feel obliged to perform extensive investigations because any person can carry a fatal disease, no matter how minor or even absent their symptoms are.

While the above position seems to discount the negative consequences of investigations, the second position acknowledges that excessive investigations can cause significant harm. Faced with a strong pressure to discover disease at the earliest opportunity and to meet patients’ demands, this second position creates a dilemma for the GPs. In this *dilemmatic position* the GPs sometimes perform investigations against their better judgement, however they also use a range of strategies to try to avoid excessive investigations. While these strategies are often demanding and not always successful, the strategy of sharing uncertainty and fallibility comes through as the most promising one.

### There is no such thing as overtesting

The GPs shared different cases where seemingly harmless symptoms turned out to be caused by a serious disease, or where a serious disease was discovered by coincidence. For example, one of the GPs had experienced a patient dying from a pulmonary embolus, which the GP failed to diagnose when the patient presented with a cough. Another GP had seen a case of serious complications from otitis media that started with mild symptoms. A nevus that was removed for cosmetic reasons turned out to be a melanoma, and a MRI taken due to shoulder pain revealed an unrelated lung cancer. These stories were used to justify a medical mind-set based on a never-ending suspicion of disease, especially for some of the trainee GPs. One of the trainees expressed this position most lucidly at the beginning of his focus group (not real but virtual names):

What do you mean? For me there is no such thing as overtesting… I do not think that I have much to contribute with in that matter… (Fredrick, group 2).

The fear of failing to discover disease was a dominant theme, as in the otitis media case mentioned above that was offered by one of the experienced GPs:

Since then it has been almost mandatory for me to check the ears of all my patients. The fright is there; what if something bad happens? Things can go really wrong when you least expect it; I have experienced that (Camilla, group 3).

### I knew that what I did was wrong

The second position found among the GPs was one of being caught between the pressure to discover disease at the earliest opportunity and the desire to fulfil patients’ demands for investigations, yet aware that excessive testing can cause harm. The GPs believed that a lack of confidence in their clinical skills made patients inclined to demand, and themselves prone to initiate high tech investigations and referrals beyond medical guidelines and best evidence.

Examples included patients with musculoskeletal complaints who demanded MRIs and did not trust the clinical judgement of their GP, even though the GP was experienced and had previously worked in orthopaedics. In another example, a patient with a chronic disease who had been thoroughly investigated demanded further examinations with gastroscopy, colonoscopy and CT of her head. This patient threatened she would go to another GP or to the emergency department unless she got what she wanted:

In the end, I was unable to resist. I knew that what I did was wrong and futile, but it was much easier to give in than to resist (Johannes, group 4).

The GPs argued that pressure from other healthcare professionals also made it harder to avoid excessive testing, as in the case of the asymptomatic patient who demanded a blood test for PSA (prostate specific antigen):

I know that the test is not indicated among asymptomatic patients, but then there is the wish to receive compliments from the urology specialist for detecting cancer at an early stage (Helen, group 1).

Harm was seen in investigations performed on the request from patients, as well as investigations initiated by the GPs themselves. The kind of harm the GPs discussed the most entailed patients experiencing enduring anxiety. The GPs were also concerned that an excessive focus on finding a medical diagnosis could prevent the patients from appropriately engaging with problems in need of other approaches:

Both she and I knew that her real problem had to do with her expectations and her anxiety. Even though I told myself that I would not investigate her with further MRIs, I was unable to resist. I continued to examine her even though it was not indicated, for her expectations were too high. In the end, it just created even more anxiety and fuelled her expectations for yet more investigations (Christine, group 4).

Harm also included false positive findings. In one example, the GP ordered blood tests for a patient who presented mild symptoms of depression and one of the liver function tests turned out to be slightly elevated. This false positive finding led to additional testing, including referrals to secondary care, which in turn fuel the patient’s anxiety.

### Sharing uncertainty and fallibility

The GPs discussed different strategies for avoiding unnecessary and harmful testing. To some extent it appeared that many GPs were already using these strategies even though they were frustrating or unsuccessful. However, these strategies were partly discussed in a hypothetical manner, as potentially useful in the future.

Firstly, the GPs described *negotiating* by offering low-tech instead of high-tech investigations, e.g. blood tests or plain radiography instead of MRI. The GPs expressed frustration about this strategy, as they did not consider the investigations they offered to be more appropriate than the ones the patients requested, and because anxiety would often be perpetuated. Still, negotiating for low-tech investigations could sometimes be a way of establishing communication about issues that the GPs thought were relevant. This was seen in the case of the patient who requested a full body MRI when presenting typical symptoms of anxiety:

I knew that I needed to negotiate with him in order to get into position, so I took a lot of blood samples to calm him down, even though it was futile. For otherwise I would never have been able to get in a position where we could have a discussion (Christopher, group 1).

The second strategy was to *put one’s foot down* to tests that the patient requested. This strategy required courage and the GPs said they had to remind themselves to trust their own professional judgment. It also appeared that they sometimes applied this strategy spontaneously out of frustration when patients requested further testing. One GP shared an example of elderly ladies who wanted yearly MRIs for their hip osteoarthritis although this was not needed from a professional point of view:

I tell them that they can´t have it, since they are not interested in surgery. I also tell them that if they want it so badly, they can pay out of their own pocket…they often accept my explanation, most do… (Helen, group 1).

The third strategy was to *widen the perspective* by discussing with the patients whether their symptoms and concerns could be related to everyday life, rather than to manage them solely in terms of biomedical investigations and treatment. The GPs’ discussion on this topic conveyed a sense of resignation as they felt patients frequently rejected such suggestions. One GP described a patient with long-standing anxiety who would turn up with different bodily complaints, including pain in her shoulder:

Maybe she knows, just as I do, that the problem really is her anxiety and the pressure she is under, both from others and from herself. Hmm … but she so strongly wants someone to say that ‘there is this tendon that has ruptured, and we can sew it together so you will be fine again’. There is this wish for something that others can fix and take responsibility for, because it is too hard to do the job yourself (Dorothy, group 3).

The fourth and final strategy entailed *sharing medical uncertainty and fallibility* with patients and colleagues. Uncertainty and fallibility included the fact that it is never possible to rule out disease, the danger of false positive findings, the increasing risk of harm as additional tests are performed, and the fact that many problems in general practice do not align with biomedical categories. The GPs discussed cases where sharing uncertainty and fallibility had enabled the physician and patient to agree on where to draw the line between meaningful and futile investigations, as in this case of lower back pain:

I told the patient that the clinical picture was stable and that a MRI could lead to further investigations and surgery. We talked about the potential risks and poor effect of surgery in her situation… in the end, together with the patient, we decided to wait and not do anything (Anne, group 1).

Another GP described how on a subsequent visit a patient had recalled her disbelief when the GP told her that she would have to manage without an additional MRI. Although the patient’s headache was not alarming to the doctor and there were the usual signs of muscular tenderness in the neck, the patient was still afraid of a cerebral tumour:

‘I went straight home,’ the patient said, ‘and told my boyfriend, ‘do you know what [my doctor] said? She said she wouldn’t refer me for another MRI. She said I would have to live with the uncertainty.’ And the boyfriend answered ‘Yes, that’s totally true' (Jane, group 4).

Although the strategy of acknowledging and sharing uncertainty and fallibility was successful in all the examples the GPs shared, they were clear that this strategy was hard to employ as it did not allow them to continue searching for disease, nor address the patient’s desire for a technical fix. As a potential remedy, the GP’s suggested sharing the burden of uncertainty and fallibility with colleagues through the context of informal discussions and in continuous educational groups.

## Discussion

### Principal findings

Our study reveals two main findings regarding GPs’ perspectives on medical overtesting. In the *categorical position* GPs act as if there is no such thing as overtesting and feel obliged to investigate each patient extensively to identify disease and accommodate the patients’ requests. In the *dilemmatic position* GPs sometimes perform excessive testing against their better judgement, but also employ different strategies for avoiding such testing. Apparently, the most promising strategy was being able to acknowledge and share medical uncertainty and fallibility with their patients.

### Strengths and weaknesses of the study

Qualitative studies are suited for exploring subjective experiences in depth and do not aim for representativeness. We cannot know to what extent the GPs we interviewed were honest in their responses or how other methodological approaches might have changed the results. However, we believe that the conflicting perspectives among our findings indicate that our results have high validity and relevance. Also, one of the main findings, i.e. ‘there is no such thing as overtesting’, goes squarely against our own preconceptions. This seems to indicate that the GPs were not overly influenced by an eagerness to please us and that our analysis was not overly influenced by our own beliefs. In the discussion below, we offer our own perspectives on the main findings. However, other interpretations are also possible.

### Findings in relation to other studies

A recent overview paper has mapped the drivers of medical overuse to five domains: the culture, the health care system, industry and technology, health care professionals and patients, and the public [12]. Our findings confirm that Norwegian GPs, along with the healthcare system, professionals and the public, sense that more medical activity is better. Our findings thus appear to echo a widespread sentiment about health and healthcare, with high expectations that professional help and technical fixes should be available for all health complaints. When disease is not discovered at its earliest stages, this is seen as a failure attributable to individual practitioners. Fear and intolerance of error of omission are common among physicians, patients and the public [13,14].

Patient-centred approaches have been promoted to curb medical paternalism. However, this can result in consumerist attitudes with patients feeling entitled to have their complaints investigated according to their own ideas about their particular medical problem. When autonomy is conflated with individual entitlement, the professional authority of GPs is undermined. And when GPs seek to put their foot down in a response to inappropriate requests from patients, this can result in emotional distress and a breakdown of trust between the GP and the patient [15].

The above leaves little room for mutual deliberation [16], promoting patients’ own salutogenic resources, watchful waiting and an acceptance that not all suffering can be eliminated. Theologian and medical ethicist Stanley Hauerwas reminds us that the very meaning of ‘patient’ includes patience and acceptance. Hauerwas argues that we need to reconsider the moral demands implied in the role of the patient, as “patients who are no longer patient in the face of illness and death cannot help but bring expectations to medicine that are corrupting” [17].

As a multitude of new medical technologies are introduced, our intellectual and organisational capacity to maximise benefits and minimise harm seems destined to lag behind [18]. Sometimes general practice research and opinion leaders are complicit in promoting the view that technologies will eliminate diagnostic uncertainty [19] and obscuring the value judgements that are involved in their use. This naïve understanding of the role of technology in the improvement of health is linked to a broader cultural reliance on technical rationality:

An implicit and almost universal assumption […] is that the problem under discussion has a technical solution. A technical solution may be defined as one that requires a change only in the techniques of the natural sciences, demanding little or nothing in the way of change in human values or ideas of morality [20].

In this cultural milieu, the assumption that medical technologies can eliminate uncertainty and the consumerist attitudes towards healthcare are becoming ‘naturalised’. The moral cost in terms of harm from medical overuse, breakdown of solidarity and the resulting underuse of healthcare for vulnerable populations are rendered invisible or irrelevant.

Despite the fact that the GPs involved in this study mainly endorsed the belief that medical actions can cause harm and that doing less sometimes is preferable to doing more, these ideas currently have little foothold in medicine and in common culture. The first idea, that medicine can harm, underpins the initial precept of the Hippocratic oath of medicine, ‘first of all, do not harm’. The gist of the second idea is that even though a certain procedure is beneficial for some patients, doing more of the same is not necessarily always best. The fundamental relevance of this idea is underpinned by the information theory insight that although increasing the amount of data is intended to reduce bias in a scientific model, this will inevitably lead to more model complexity, variance and error [21]. In other words, this idea can be interpreted as an extension of the law of diminishing returns combined with an acknowledgment of the existence of harm [22].

Sixty years ago, the American sociologist Renee Fox observed how the training of medical students and junior doctors centred on different strategies for managing uncertainty. Revisiting and reassessing the role of uncertainty in medicine forty years later, Fox commented on how technological progress has reshaped rather than eliminated uncertainty:

Scientific, technological and clinical advantages change the content of medical uncertainty and alter its contours, but they do not drive it away. Furthermore, although medical progress dispels some uncertainties, it uncovers others that were not formerly recognized, and it may even create new areas of uncertainty that did not previously exist [23].

Malterud et al have recently suggested that it is time for general practice “to develop theoretical, clinical, and practical strategies for embracing – not simply tolerating – uncertainty, instead of unsuccessfully trying to eradicate or suppress it” [24]. Our study underscores the timeliness of this proposal. Along the lines of “collaborative engagement” that Sommers and Launer have introduced [25], we suggest that it is a core skill for primary care physicians to manage and share uncertainty with patients and society. This might give better results than colluding with the myth that the more medical activities we pursue, the more certain we can be of the patients' good health.

## Conclusions

A strong pressure to avoid errors of omission and to accommodate patients’ requests leads GPs in Norway to pursue investigations resulting in patient harm. Sharing fallibility and uncertainty with patients and colleagues seems to be a promising strategy to avoid overtesting.
